# Sterol Regulatory Element-Binding Protein-1c Regulates Inflammasome Activation in Gingival Fibroblasts Infected with High-Glucose-Treated *Porphyromonas gingivalis*

**DOI:** 10.3389/fcimb.2016.00195

**Published:** 2016-12-26

**Authors:** Hsing-Chun Kuo, Li-Ching Chang, Te-Chuan Chen, Ko-Chao Lee, Kam-Fai Lee, Cheng-Nan Chen, Hong-Ren Yu

**Affiliations:** ^1^Department of Nursing, Chang Gung University of Science and Technology (CGUST)Chiayi, Taiwan; ^2^Research Center for Industry of Human Ecology and Research Center for Chinese Herbal Medicine, College of Human Ecology, Chang Gung University of Science and Technology (CGUST)Taoyuan, Taiwan; ^3^Chronic Diseases and Health Promotion Research Center, Chang Gung University of Science and Technology (CGUST)Chiayi, Taiwan; ^4^Department of Dentistry, Chang Gung Memorial HospitalChiayi, Taiwan; ^5^Division of Nephrology Kaohsiung Chang Gung Memorial Hospital and Chang Gung University College of MedicineKaohsiung, Taiwan; ^6^Division of Colorectal Surgery, Department of Surgery, Chang Gung Memorial Hospital - Kaohsiung Medical CenterKaohsiung, Taiwan; ^7^Department of Pathology, Chang Gung Memorial HospitalChiayi, Taiwan; ^8^Department of Biochemical Science and Technology, National Chiayi UniversityChiayi, Taiwan; ^9^Department of Pediatrics, Chang Gung Memorial Hospital – Kaohsiung Medical CenterKaohsiung, Taiwan; ^10^Graduate Institute of Clinical Medical Science, Chang Gung University College of MedicineKaohsiung, Taiwan

**Keywords:** *Porphyromonas gingivalis*, gingival fibroblasts, high glucose, NLRP3, SREBP-1c

## Abstract

**Background:** *Porphyromonas gingivalis* is a major bacterial species implicated in the progression of periodontal disease, which is recognized as a common complication of diabetes. The interleukin (IL)-1β, processed by the NLR family pyrin domain containing 3 (NLRP3) inflammasome, has been identified as a target for pathogenic infection of the inflammatory response. However, the effect of *P. gingivalis* in a high-glucose situation in the modulation of inflammasome activation in human gingival fibroblasts (HGFs) is not well-understood.

**Methods:** *P. gingivalis* strain CCUG25226 was used to study the mechanisms underlying the regulation of HGF NLRP3 expression by the infection of high-glucose-treated *P. gingivalis* (HGPg).

**Results:** HGF infection with HGPg increases the expression of IL-1β and NLRP3. We further demonstrated that the upregulation of sterol regulatory element-binding protein (SREBP)-1c by activation of the Akt and p70S6K pathways is critical for HGPg-induced NLRP3 expression. We showed that the inhibition of Janus kinase 2 (JAK2) blocks the Akt- and p70S6K-mediated SREBP-1c, NLRP3, and IL-1β expression. The effect of HGPg on HGF signaling and NLRP3 expression is mediated by β1 integrin. In addition, gingival tissues from diabetic patients with periodontal disease exhibited higher NLRP3 and SREBP-1c expression.

**Conclusions:** Our findings identify the molecular pathways underlying HGPg-dependent NLRP3 inflammasome expression in HGFs, providing insight into the effect of *P. gingivalis* invasion in HGFs.

## Introduction

Periodontal diseases, the most common chronic inflammatory diseases in adults, are characterized by bacteria-induced loss of connective tissues within the periodontium and the destruction of alveolar bone support (Hajishengallis, 2015). Infection of *Porphyromonas gingivalis*, a gram-negative oral anaerobic bacterium, has been proposed as the primary etiological pathogen associated with increased risk of periodontal breakdown and disease recurrence (Darveau, 2010; Gaddis et al., 2011; Ji et al., 2015). Moreover, it has been reported that the risk of periodontitis is significantly higher in individuals with diabetes than in normal subjects (Nibali et al., 2007). Our previous study reported that when cultured under high-glucose (HG) conditions, the invasion efficiency of HG-treated *P. gingivalis* (HGPg) into human gingival fibroblasts (HGFs) and the expression of intercellular adhesion moleculae-1 was significantly increased (Chang et al., 2013). Furthermore, several reports have demonstrated that non-surgical periodontal therapies can improve the condition of patients with high blood glucose (Faria-Almeida et al., 2006; Lin et al., 2012). Periodontal treatment with antibiotics also has significantly positive effects on glycemic control in diabetic patients (Bharti et al., 2013).

Fimbriae of *P. gingivalis* play critical roles in mediating the initiation and development of periodontal disease. *P. gingivalis* can adhere to oral surfaces or mucosa predominantly by peritrichous fimbriae (Yoshimura et al., 2009). The strains with different *fimA* genotypes contribute to distinct *P. gingivalis* virulence and play a critical role in regulating the development of periodontal disease (Kato et al., 2007). Our previous study reported that the fimA mRNA was markedly upregulated in HGPg compared to normal glucose-treated *P. gingivalis* (NGPg), and the invasion efficiency of HGPg to HGFs was also significantly increased (Chang et al., 2013). *P. gingivalis* infection and the host inflammatory response are necessary for the progression of periodontal disease, especially in patients with diabetes. However, the precise roles and detailed mechanisms of host-mediated inflammation by HGPg infection have not been determined.

The inflammasome, a multiprotein complex that triggers the production of mature IL-1β in response to intracellular stress signals, plays a key role in innate immunity (Lamkanfi and Dixit, 2014). The best characterized inflammasome is the NOD-like receptor protein 3 (NLRP3) inflammasome. Upon activation, NLRP3 leads to the activation of proteinase Caspase-1, which in turn processes pro-IL-1β into its mature form (Jo et al., 2016). There is growing evidence of a relationship between diabetes and inflammasome activation (Lee et al., 2013). The lack of inflammasome components has provided evidence that NLRP3 activation is a key mechanism that induces systemic inflammation and the development of insulin resistance (Dixit, 2013). Although the connections between hyperglycemia and inflammation have been extensively characterized (Chen et al., 2011a; Chen T. C. et al., 2014), the molecular mechanisms responsible for NLRP3 expression after infection with *P. gingivalis* cultured under HG conditions remains unclear.

Sterol regulatory element binding protein-1c (SREBP-1c), a key lipogenic transcription factor, regulates cholesterol and fatty acid metabolism (Wang et al., 2015). SREBP-1c plays an important role in the inflammatory response; it is a critical regulator in the induction of inflammatory cytokine expression involved in lipid metabolism (Li et al., 2015). High levels of SREBP-1c expression and nuclear accumulation have been observed in diabetic mouse models (Laplante and Sabatini, 2010). Unfortunately, no studies have determined the relationship among *P. gingivalis* infection, SREBP-1c expression and inflammasome activation.

HGFs, the major cell type found in the periodontal connective tissue, can upregulate inflammatory mediators in response to pathogen-associated stimuli (Chang et al., 2013). It has been clarified that HGFs regulate inflammatory reactions in the development of periodontal disease. Furthermore, diabetes is connected with a higher risk of severe periodontal disease, and poorly controlled diabetes is a major contributor to poorer periodontal health (Preshaw et al., 2012). The mechanisms that link diabetes and periodontal disease have not been completely evaluated, but they involve an immune system response to infection and inflammation (Preshaw et al., 2012). In addition, it has been reported that the fimbriated *P. gingivalis* can activate β forms of integrins, leading to the manipulation of host cell function and causing disease (Hajishengallis et al., 2009; Zhang et al., 2013). These signals may lead to the activation of Janus kinase (JAK) pathways. The purpose of this study was to investigate the molecular mechanism that regulates the expression of HGF NLRP3 inflammasome by HGPg. The results demonstrated that the NLRP3 inflammasome activation induced by HGPg is mediated through the β1 integrin, the intracellular signaling cascades Akt and p70S6K, and the nuclear SREBP-1c transcription factors.

## Methods

### Materials

All culture materials were purchased from Gibco (Grand Island, NY). LY294002 (PI3K/Akt inhibitor) and rapamycin (p70S6K inhibitor) were purchased from Calbiochem (La Jolla, CA). Mouse monoclonal antibodies (mAB) against SREBP-1c, NLRP3, IL-1β, pro-IL-1β, caspase-1, pro-caspase-1, phospho-Akt, and Akt were purchased from Santa Cruz Biotechnology (Santa Cruz, CA). Rabbit polyclonal antibodies against phospho-p70S6K, p70S6K, phospho-JAK2, and JAK2 were purchased from Cell Signaling Technology (Beverly, MA). SREBP-1c, NLRP3, JAK2 siRNA and control siRNA were purchased from the National RNAi Core Facility in Academic Sinica, Taipei, Taiwan. The integrin β1-siRNA were purchased from Invitrogen (Carlsbad, CA). *P. gingivalis* lipopolysaccharide (Pg-LPS) was purchased from InvivoGen (San Diego, CA). Other chemicals of reagent grade were obtained from Sigma (St. Louis, MO).

### Bacterial strains and growth conditions

Periodontal pathogens *P. gingivalis* strain CCUG25226 and *Fusobacterium nucleatum* strain CCUG51781 were purchased from the Bioresources Collection and Research Center of the Food Industry Research and Development Institute (Hsinchu, Taiwan). These bacteria were inoculated anaerobically in brain-heart infusion (BHI) broth in 37°C supplemented with 0.5% yeast extract, 5 μg/mL hemin and 1 μg/mL vitamin K3. Pathogens grown were monitored by recording the optical density at 660 nm.

For studying the effect of glucose on *P. gingivalis* and *F. nucleatum*, and the further effect of HGPg on HGF NLRP3 expression, *P. gingivalis* was cultured in BHI medium containing 5 (normal glucose, NG) or 25 (HG) mmol/L glucose. Then *P. gingivalis* was collected and defined as NGPg and HGPg. For the infection assay, the indicated multiplicity of infection (MOI) of NGPg or HGPg was added to HGFs according to different experimental treatments.

### Cell culture

HGFs were obtained from ScienCell Research Laboratories (San Diego, CA). Cells were incubated at 37°C in DMEM median supplemented with 10% fetal bovine serum (FBS), penicillin (100 U/mL) and streptomycin (100 μg/mL), and grown in a humidified atmosphere containing 5% CO_2_. HGFs from passage levels 4–6 were used in this study.

### Invasion assays

HGFs were infected with bacteria at 37°C with indicated MOI. After 1 h incubation, HGFs were washed with PBS and then cultured for another 2 h to kill extracellular *P. gingivalis* in medium containing gentamicin and metronidazole. Cells were then washed and lysed in 0.1% Triton X-100, and plated on blood agar plates. Bacteria present in the lysates were titered, representing the number of bacteria intracellularly. Invasion levels were calculated as the number of *P. gingivalis* surviving divided by the total number of bacteria.

### Purification of fimbriae from *P. gingivalis*

Fimbriae form NGPg or HGPg were purified as described previously (Chen et al., 2011b). The purified fimbriae were tested for agglutinating activity with erythrocytes. The protein content was determined and the fractions showing agglutinating activity were subjected to SDS-PAGE and western blot analysis.

### Real-time quantitative PCR

For detecting the levels of HGF mRNA expression, real-time PCR was performed, and products were detected using an ABI Prism 7900HT with the FastStart DNA SYBR Green I kit (Roche Diagnostics GMbH, Mannheim, Germany). The designed primers in this study were SREBP-1c forward primer, 5′-GTGAC ATGCA GCACC TCCTG-3′; SREBP-1c reverse primer, 5′-TCCAT GGTGA TCTCT CCTCA-3′; 18S rRNA forward primer, 5′-CGGCG ACGAC CCATT CGAAC-3′, 18S rRNA reverse primer, 5′-GAATC GAACC CTGAT TCCCC GTC-3′; NLRP3 forward primer, 5′-AAAAG ACTCA TCCGT GTGCC-3′; NLRP3 reverse primer, 5′-TTCCT GGCAT ATCAC AGTGG-3′; IL-1β forward primer, 5′-AAACA GATGA AGTGC TCCTT CCAGG-3′; IL-1β reverse primer, 5′-TGGAG AACAC CACTT GTTGC TCCA-3′. fimA forward primer, 5′-CAGCA GGAAG CCATC AAATC-3′; fimA reverse primer, 5′-CAGTC AGTTC AGTTG TCAAT-3′; 16S rRNA forward primer, 5′-TGTAG ATGAC TGATG GTGAA A-3′; and 16S rRNA reverse primer, 5′-ACTGT TAGCA ACTAC CGATG T-3′. Quantification was performed using the 2^−ΔΔCt^ method. All samples were measured in duplicate. The average value of both duplicates was used as the quantitative value.

### Western blot analysis

Cells were lysed with a buffer containing 1% NP-40, 0.5% sodium deoxycholate, 0.1% SDS, and a protease inhibitor mixture (PMSF, aprotinin, and sodium orthovanadate). The total cell lysate (50 μg of protein) was separated by SDS-polyacrylamide gel electrophoresis (PAGE) (12% running, 4% stacking) and analyzed using the designated antibodies and the Western-Light chemiluminescent detection system (Bio-Rad, Hercules, CA) (Huang et al., 2016).

### IL-1β enzyme-linked immunosorbent assay (ELISA)

The levels of IL-1β in the media were determined by using sandwich ELISA kit (sensitivity 18 pg/mL; R&D) according to the manufacturer's protocols (R & D Systems, Minneapolis, MN) (Tseng et al., 2016).

### siRNA transfection

For siRNA transfection, HGFs were transfected with the specific siRNAs or control siRNA by using an RNAiMAX transfection kit (Invitrogen, Carlsbad, CA).

### Reporter gene construct and luciferase assays

SREBP-1c promoter construct contain −1470/+90 of SREBP-1c 5′-flanking DNA linked to the firefly luciferase reporter gene of plasmid pGL4 (Promega, Madison, WI). DNA plasmids at a concentration of 1 mg/ml were transfected into HGFs by using Lipofectamine (Gibco). The pSV-β-galactosidase plasmid was cotransfected to normalize the transfection efficiency.

### Subjects and collection of gingival tissue samples

All study protocols were in accordance with the Declaration of Helsinki and were approved by the Medical Ethics Committee of Chang Gung Memorial Hospital (No. 104–1541C), and all patients provided written informed consent before enrollment. Ten patients with both periodontal disease and type 2 diabetes, and 10 control patients with periodontal disease alone were recruited in this study. The diabetic patients had a mean (±SEM) age of 47.6 ± 5.7 years, fasting glucose of 11.7 ± 1.1 mmol/L, a body mass index (BMI) of 29.6 ± 3.1 kg/m^2^, and hemoglobin A1_C_ (HbA1c) of 7.4 ± 1.3%. In addition, the control subject group had a mean (±SEM) age of 46.3 ± 6.4 years, fasting glucose of 4.7 ± 0.1 mmol/L, a BMI of 25.8 ± 1.6 kg/m^2^, and HbA1c of 4.4 ± 0.6%. None of the control subjects had cardiac, renal, or pulmonary decompensated diseases, or other infectious or inflammatory situations. Subjects who smoked cigarettes or used alcohol, non-steroidal anti-inflammatory drugs, corticosteroids, anticoagulant drugs, or hormonal replacement therapy were excluded. Patients had not been treated for periodontitis over the previous 2 years and had taken no antibiotics in the 6 months preceding surgery. Gingival samples were obtained from subjects undergoing surgery to treat periodontitis.

### Immunohistochemical (IHC) analysis of SREBP-1c and NLRP3 expression

Gingival tissue samples were frozen immediately after surgery in liquid nitrogen and stored at −80°C. Serial paraffin sections of biopsies were cut 5 μm thick, and IHC assays was performed using the immunoperoxidase staining. For inhibition of non-specific binding, the tissue sections were incubated with normal goat serum and non-fat dry milk. Subsequently, the sections were incubated at 4°C with primary antibodies against SREBP-1c and NLRP3, respectively. Sections stained with normal mouse IgG as primary antibody were used as a negative control (Kuo et al., 2016). Sections were then reacted with secondary antibody, followed by incubation with avidin-biotin-peroxidase complex with the addition of DAB (3,3′-diaminobenzidine tetrahydrochloride).

### Statistical analysis

The results are expressed as mean ± standard error of the mean (SEM). Statistical analysis was determined by using an independent Student *t*-test for two groups of data and analysis of variance (ANOVA) followed by Scheffe's test for multiple comparisons. *P*-values less than 0.05 were considered significant (Lu et al., 2016).

## Results

### HGPg infection induced the expression of IL-1β in HGFs

**Figure 1:**
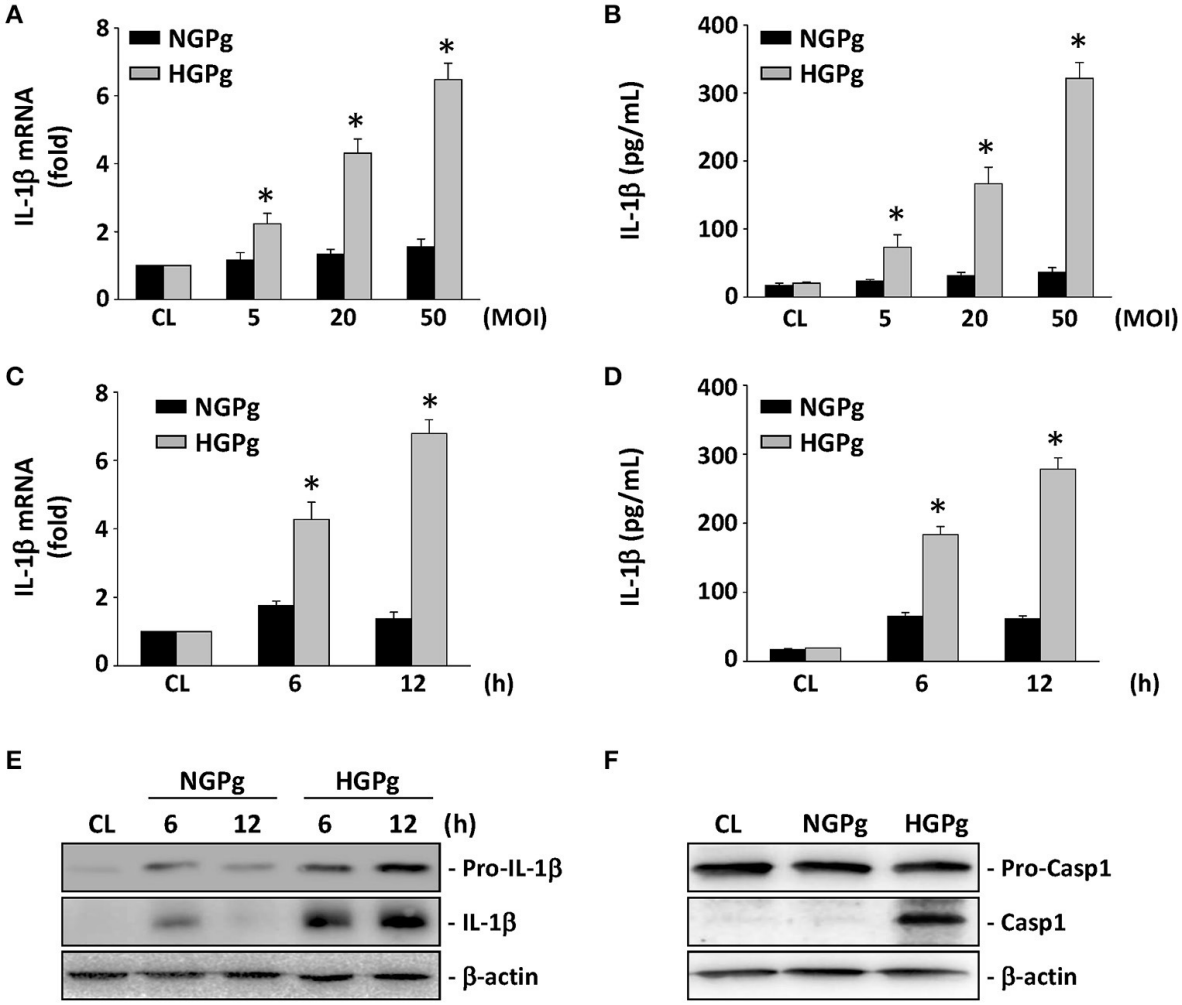
**Induction of IL-1β expression in HGFs infected with ***P. gingivalis*****. RNA samples were isolated at the indicated MOI or times. All bar graphs represent folds of control cells (CL) normalized to 18S rRNA by real-time PCR analysis **(A,C)**. IL-1β secretion in medium was determined by ELISA analyses **(B,D)**. **(A,B)** Cells were infected by NGPg or HGPg at various MOI for 6 h, or **(C,D)** infected with NGPg or HGPg at 20 MOI for 6 and 12 h. Data are shown as mean ± standard error of the mean (SEM). ^*^P < 0.05 vs. CL HGFs or HGFs infected with NGPg. **(E,F)** The expression of pro-IL-1β and IL-1β **(E)**, or pro-caspase-1 and caspase-1 **(F)** in HGF cell lysate after HGPg infection for the times indicated was determined using Western blotting.

To demonstrate that HGPg infection upregulates the expression and secretion of IL-1β, the HGFs were infected with different MOI of HGPg (vs. NGPg) for 6 h. As shown in Figures 1A,B, the induction of IL-1β mRNA expression (Figure 1A) and IL-1β protein secretion in medium (Figure 1B) by HGPg infection was MOI-dependent. The time courses determined for the IL-1β mRNA levels revealed an increase after 6 and 12 h of HGPg infection (Figure 1C). HGPg also caused significant upregulation in the secretion of IL-1β into medium at 6 and 12 h after infection in HGFs (Figure 1D). In addition, the cytoplasmic pro-IL-1β and mature IL-1β protein levels were increased in HGFs infected with HGPg (Figure 1E). Upon inflammasome formation, pro-caspase-1 underwent autocleavage to produce caspase-1. Western blot analysis showed that active caspase-1 was observed at 6 h after HGPg infection, suggesting inflammasome activation in HGFs (Figure 1F).

We further investigated the invasion efficiency and IL-1β mRNA expression of normal or HG-cultured HGFs infected with NGPg or HGPg. HGPg infected HGFs much more readily in both normal and HG-cultured HGFs (Figure S1A). The IL-1β mRNA expression in normal or HG-culture HGFs was also significantly increased when infected with HGPg, whereas NGPg infection only had a marginal effect on IL-1β mRNA expression in HG-cultured HGFs (Figure S1B). To determine whether IL-1β mRNA expression was dependent on specific *P. gingivalis* carrying fimA, HGFs were infected with another periodontal pathogen *F. nucleatum* cultured in NG (NGFn) or HG (HGFn) conditions. As shown in Figure S2, NGFn and HGFn caused a similar effect on IL-1β expression in HGFs. HGPg was used as a positive control.

The *fimA* mRNA expression in NGPg and HGPg was further detected by real-time PCR. As shown in Figure S3A, the expression levels of *fimA* mRNA were significantly higher in HGPg. To determine the protein expression levels of fimbriae in NGPg and HGPg, fimbriae were purified and detected by Western blotting. Our results revealed that FimA protein levels were also significantly increased in HGPg (Figure S3B). Stimulation of HGFs with purified fimbriae from HGPg, or co-stimulation of HGFs with Pg-LPS and purified fimbriae from HGPg, significantly increased IL-1β mRNA expression, whereas co-stimulation with Pg-LPS and purified fimbriae from NGPg had a minor effect on IL-1β expression in HGFs (Figure S3C).

### Infection of the HGFs by HGPg induced NLRP3 and SREBP-1c expression

**Figure 2:**
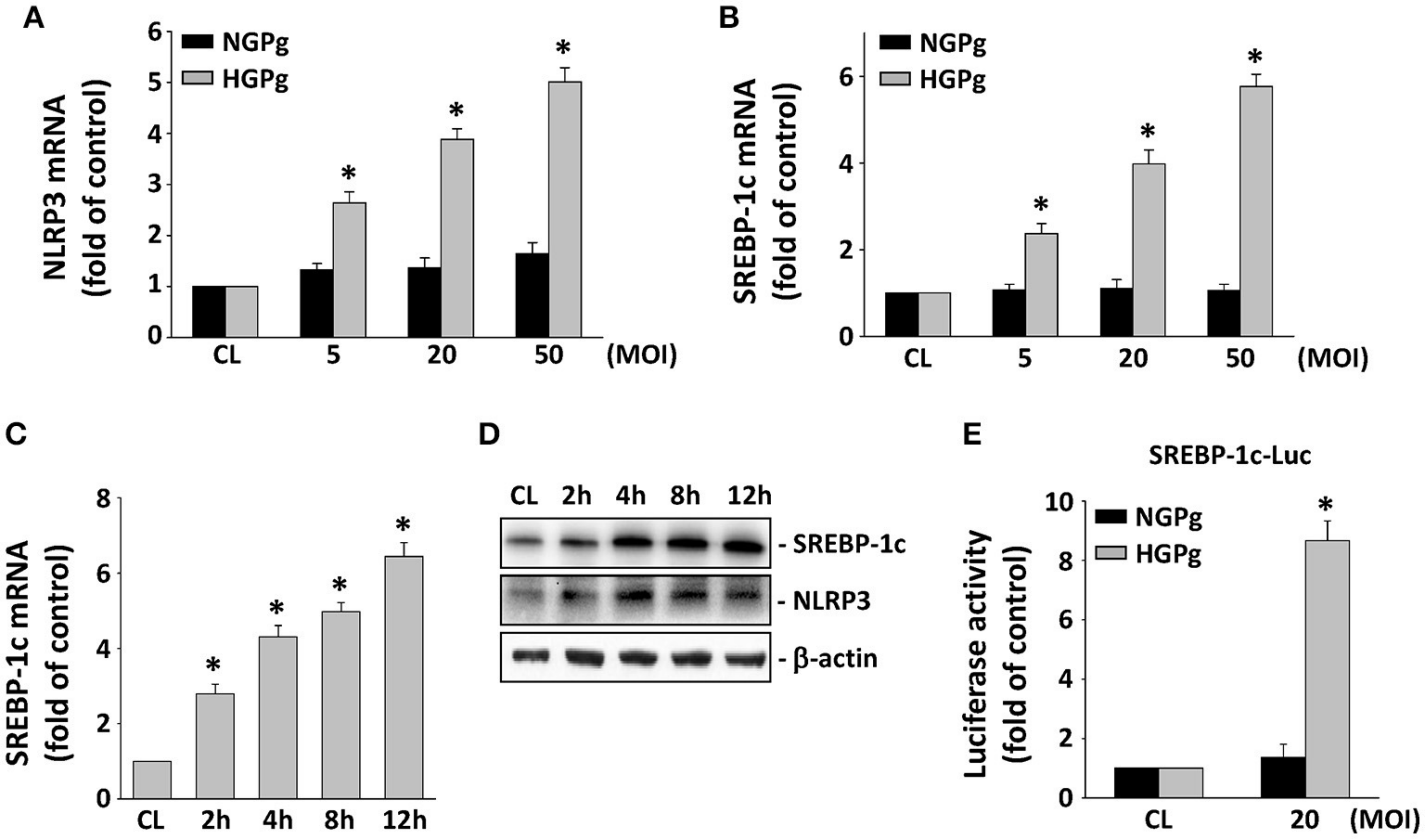
**Induction of NLRP3 and SREBP-1c expression in HGFs infected with HGPg**. RNA samples were isolated at the indicated MOI or times. All bar graphs represent folds of control cells (CL) normalized to 18S rRNA by real-time PCR analysis **(A–C)**. **(A,B)** Cells were infected by NGPg or HGPg at various MOI for 4 h, or **(C)** infected with HGPg at 20 MOI for times indicated. **(D)** The expression of mature 68 kD SREBP-1c and NLRP3 in HGF cell lysate after HGPg infection for the times indicated was determined using Western blotting. **(E)** HGFs were cotransfected with SREBP-1c-Luc and infected with NGPg or HGPg at 20 MOI for 4 h. SREBP-1c promoter activity was measured by luciferase assay normalized to β-galactosidase activity. Data are shown as mean ± standard error of the mean (SEM). ^*^P < 0.05 vs. CL HGFs or HGFs infected with NGPg.

The effects of HGPg infection on the mRNA expression of NLRP3 in HGFs were studied using infected cells with *P. gingivalis* grown on NG or under HG conditions. Consistent with our hypothesis, HGF infection with HGPg induced MOI-dependent NLRP3 gene expression (Figure 2A). NGPg infection failed to induce significant NLRP3 expression, even at a high MOI, suggesting that NGPg infection is insufficient to trigger inflammasome activation. The infection of HGFs by HGPg also caused significant increases in the SREBP-1c gene expression in an MOI-dependent manner (Figure 2B). The induction of SREBP-1c mRNA expression by HGPg was time-dependent (Figure 2C). HGPg infection also induced an increase in NLRP3 and mature 68 kD SREBP-1c protein expression in HGFs in a time-dependent manner (Figure 2D). In addition, culturing of the HGFs with HGPg increased the luciferase activity 6.3-fold compared with NGPg-infected HGFs after normalization with a transfection control (Figure 2E).

### Effects of SREBP-1c on HGPg-induced NLRP3 and IL-1β expression

**Figure 3:**
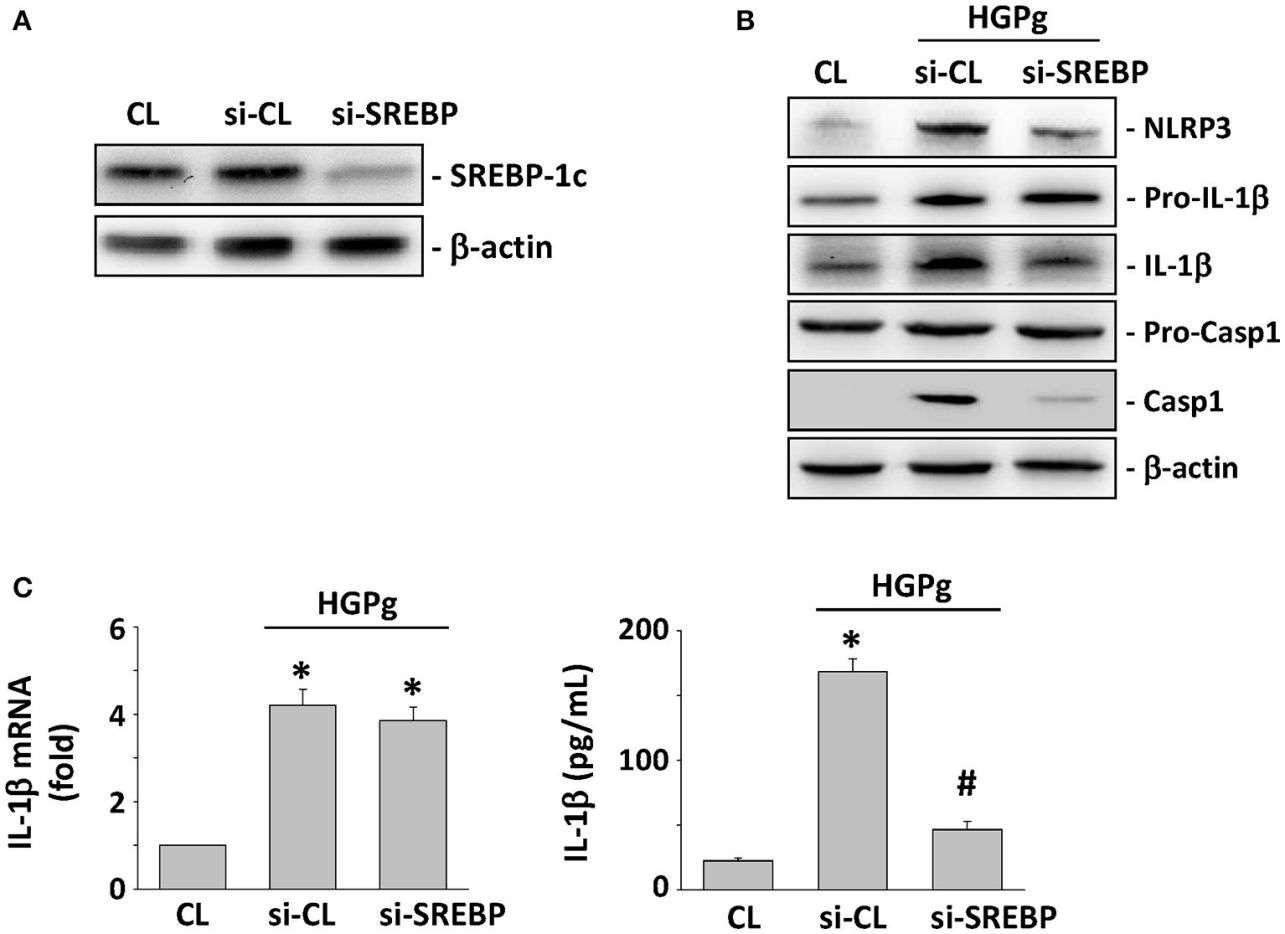
**Effects of SREBP-1c on HGPg-induced NLRP3 and IL-1β expression. (A)** The gene silencing efficiency of 48 h transfection of siRNA on SREBP-1c levels of HGFs. After 48 h of transfection, protein was isolated and the SREBP-1c expression was analyzed by Western blotting. **(B,C)** HGFs were kept as CL or infected by HGPg (20 MOI). Before being kept as CL or infected by HGPg, HGFs were transfected with control siRNA (si-CL), or a specific siRNA of SREBP-1c (si-SREBP). **(B)** The expression of NLRP3, pro-IL-1β, IL-1β, pro-caspase-1, and caspase-1 in HGF cell lysate after HGPg infection was determined using Western blotting. **(C)** IL-1β secretion in medium was determined by ELISA analyses. ^*^p < 0.05 vs. control HGFs.^#^*p* < 0.05 vs. si-SREBP-1c-transfected cells with HGPg infection.

To investigate the role of SREBP-1c on HGPg-induced NLRP3 and IL-1β expression, HGFs were incubated with specific siRNA for SREBP-1c before infection with HGPg. The SREBP-1c-specific siRNAs (compared with the control siRNA) caused a 90% reduction in SREBP-1c protein expression (Figure 3A). After SREBP-1c expression was suppressed using siRNA, the expression of NLRP3, mature IL-1β, and active caspase-1 in the HGPg-infected HGFs was significantly decreased compared to that in the control siRNA-transfected HGFs, while pro-IL-1β and pro-caspase-1 were unaltered (Figure 3B). The IL-1β mRNA expression levels in the HGPg-infected HGFs did not change between the control siRNA and the SREBP-1c siRNA-treated cells (Figure 3C, left panel). However, the concentration of IL-1β in the medium was reduced due to the inhibition of SREBP-1c expression (Figure 3C, right panel), since pro-IL-1β development into mature IL-1β is regulated by NLRP3 activation. These results indicate that the expression of SREBP-1c in HGFs may play a critical role in regulating NLRP3 inflammasome expression infected by HGPg.

### Akt and p70S6K activation regulate SREBP-1c-mediated NLRP3 and IL-1β expression

**Figure 4:**
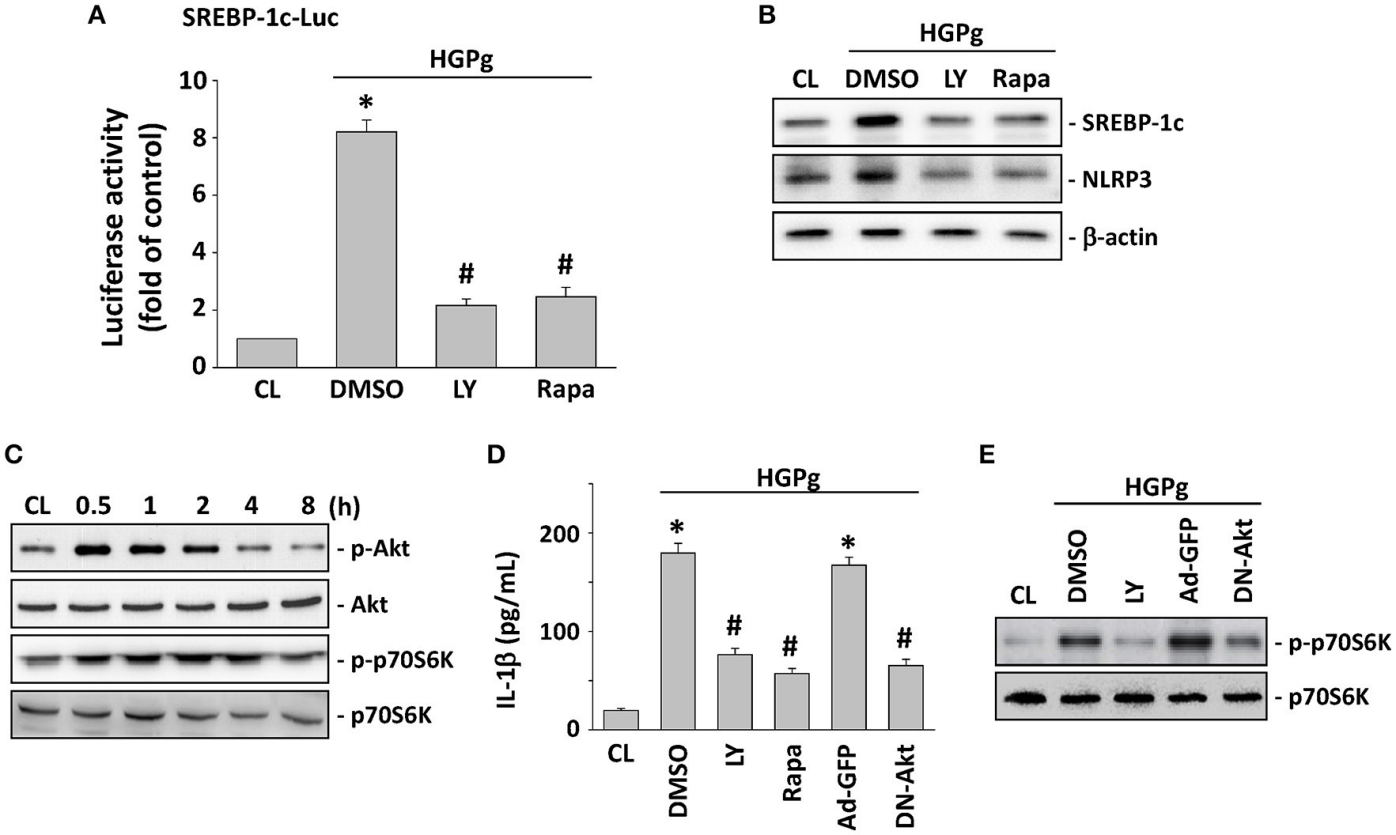
**SREBP-1c regulates NLRP3 and IL-1β expression through Akt and p70S6K activation**. HGFs were kept as CL or infected by HGPg (20 MOI). Before being kept as CL or infected by HGPg, HGFs were pretreated with LY294002 (LY) or rapamycin (rapa) individually for 1 h, or infected with adenovirus expressing the GFP (ad-GFP) or dominant-negative (DN)-Akt. **(A)** SREBP-1c-Luc activity determined in HGFs pretreated with LY or rapa individually, and then infected with HGPg for 4 h. **(B)** The expression of NLRP3 and SREBP-1c in HGF cell lysate after HGPg infection was determined using Western blotting. **(C)** HGFs were kept as CL or stimulated with HGPg for the times indicated, and the phosphorylation of Akt and p70S6K was determined by Western blotting. **(D)** IL-1β secretion in medium was determined by ELISA analyses. The results are shown as mean ± SEM. ^*^P < 0.05 vs. CL. ^#^*P* < 0.05 vs. vehicle control (DMSO) or Ad-GFP with HGPg infection. **(E)** The phosphorylation of p70S6K in HGF cell lysate after HGPg infection was determined using Western blotting.

The PI3K/Akt/p70S6K pathway has been shown to regulate a number of cellular processes, including gene expression and inflammation (Xie et al., 2014). To investigate the involvement of PI3K/Akt and p70S6K in the modulation of SREBP-1c and NLRP3 expression by HGPg infection, HGFs were incubated with specific inhibitors for PI3K/Akt (LY294002) and p70S6K (rapamycin) for 1 h before and during infection with HGPg. Pretreatment of the HGFs with LY294002 and rapamycin resulted in a marked inhibition of HGPg-induced SREBP-1c promoter activity (Figure 4A). LY294002 and rapamycin were also found to significantly inhibit the HGPg-induced protein expression of SREBP-1c and NLRP3 (Figure 4B). The levels of phosphorylated Akt and p70S6K in HGFs increased significantly after 30 min of HGPg infection (Figure 4C). To further confirm the involvement of Akt and p70S6K in the modulation of IL-1β secretion into medium by HGPg stimulation, we examined the effects of cells pretreated with inhibitors or adenovirus expressing the dominant-negative (DN) Akt on HGPg-induced IL-1β secretion. HGPg-induced IL-1β in medium was inhibited by cells pretreated with LY294002 and rapamycin, as was infection with DN-Akt, but not by treatment with vehicle controls (Figure 4D). Furthermore, LY294002 and DN-Akt inhibited the HGPg-induced phosphorylation of p70S6K (Figure 4E). These results indicate that Akt is an upstream regulator for HGPg-induced signaling pathways in HGFs.

### JAK2 is required for Akt and p70S6K phosphorylation and SREBP-1c-mediated NLRP3 and IL-1β expression

**Figure 5:**
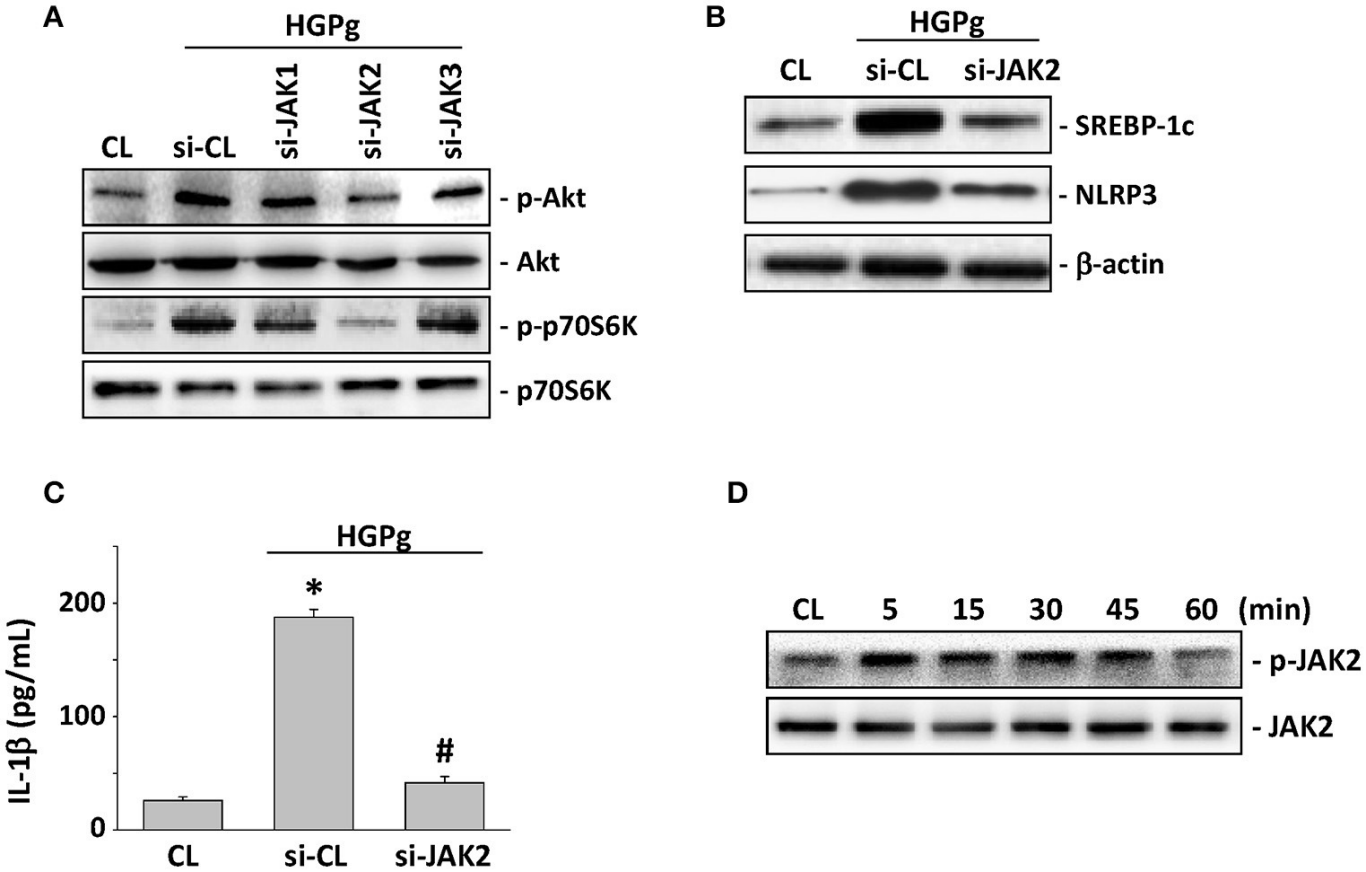
**JAK2 is required for Akt activation and SREBP-1c-mediated NLRP3 and IL-1β expression**. HGFs were kept as CL or infected by HGPg (20 MOI). Before being kept as CL or infected by HGPg, HGFs were transfected with si-JAK1, 2, or 3. **(A)** The phosphorylation of Akt and p70S6K in HGF cell lysate after HGPg infection was determined using Western blotting. **(B)** The expression of NLRP3 and SREBP-1c in HGF cell lysate after HGPg infection was determined using Western blotting. **(C)** IL-1β secretion in medium was determined by ELISA analyses. The results are shown as mean ± SEM. ^*^P < 0.05 vs. CL. ^#^*P* < 0.05 vs. si-CL-transfected HGFs with HGPg infection. **(D)** The phosphorylation of JAK2 in HGF cell lysate after HGPg infection was determined using Western blotting.

The JAK family of protein tyrosine kinases has been shown to induce Akt phosphorylation and SREBP-1 expression (Wu et al., 2013). To evaluate the role of JAK family members in HGPg-induced inflammasome activation, HGFs were transfected with JAK1, 2, or 3-siRNA and followed by infection with HGPg. The HGPg-induced Akt and p70S6K phosphorylation were significantly suppressed by the inhibition siRNA of JAK2 (Figure 5A). In addition, the HGPg-induced protein expression of SREBP-1c and NLRP3 (Figure 5B) and the secretion of IL-1β in medium (Figure 5C) were also markedly inhibited by specific JAK2-siRNA. Stimulation of HGFs with HGPg resulted in an increase of JAK2 Tyr1007/1008 phosphorylation in a time-dependent manner. The response peaked at 5 min and declined after 60 min of infection (Figure 5D).

### β1 integrin is required for HGPg-induced NLRP3 expression

**Figure 6:**
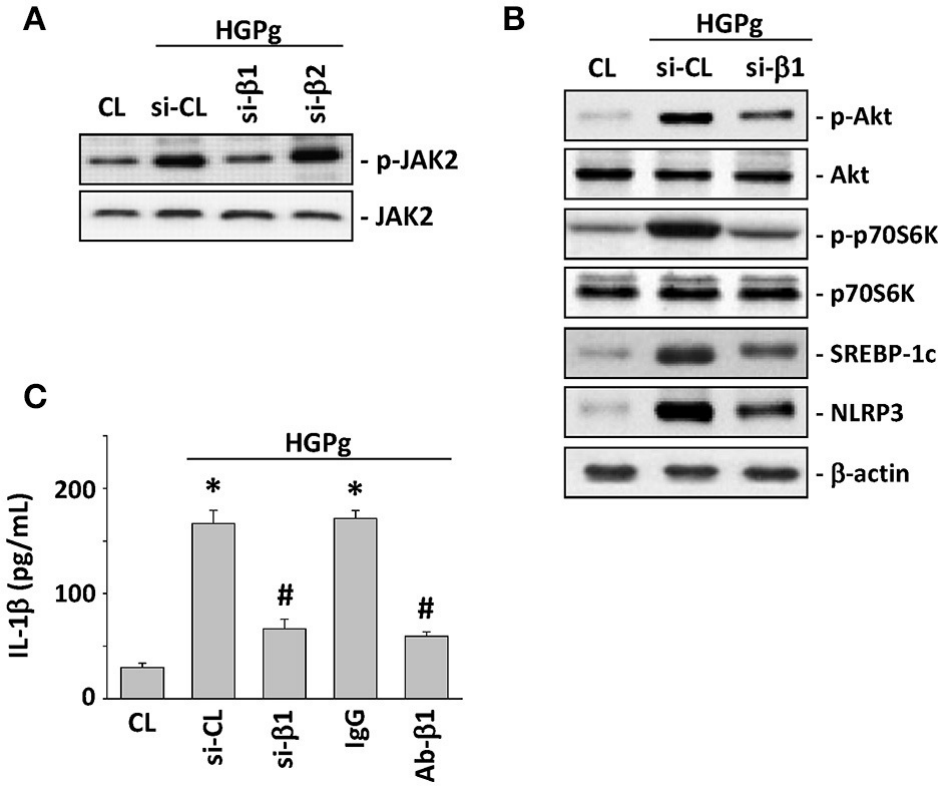
**β1 integrin is required for HGPg-induced NLRP3 expression**. HGFs were kept as CL or infected by HGPg (20 MOI). Before being kept as CL or infected by HGPg, HGFs were transfected with si-integrin β1 or β2, or pretreated with neutralizing antibody against integrin β1. **(A)** The phosphorylation of JAK2 in HGF cell lysate after HGPg infection was determined using Western blotting. **(B)** The phosphorylation of Akt and p70S6K, and the expression of NLRP3 and SREBP-1c in HGF cell lysate after HGPg infection, were determined using Western blotting. **(C)** IL-1β secretion in medium was determined by ELISA analyses. The results are shown as mean ± SEM. ^*^P < 0.05 vs. CL. ^#^*P* < 0.05 vs. si-CL-transfected or IgG-treated HGFs with HGPg infection.

It has been reported that *P. gingivalis* fimbriae can bind and activate integrins, and further affect host cell gene expression (Zhang et al., 2013). To assess the role of β forms of integrins in HGPg-stimulated HGF function, we evaluated the effects of integrin β1 and β2-siRNA on HGF infected with HGPg. The HGPg-induced JAK2 phosphorylation was significantly reduced by integrin β1-siRNA (Figure 6A). In addition, the HGPg-induced phosphorylation of Akt and p70S6K and the protein expression of SREBP-1c and NLRP3 (Figure 6B), as well as the secretion of IL-1β in medium (Figure 6C), were also decreased in HGFs transfected with integrin β1-siRNA.

### Higher SREBP-1c and NLRP3 expression in human periodontitis tissues from diabetes patients

**Figure 7:**
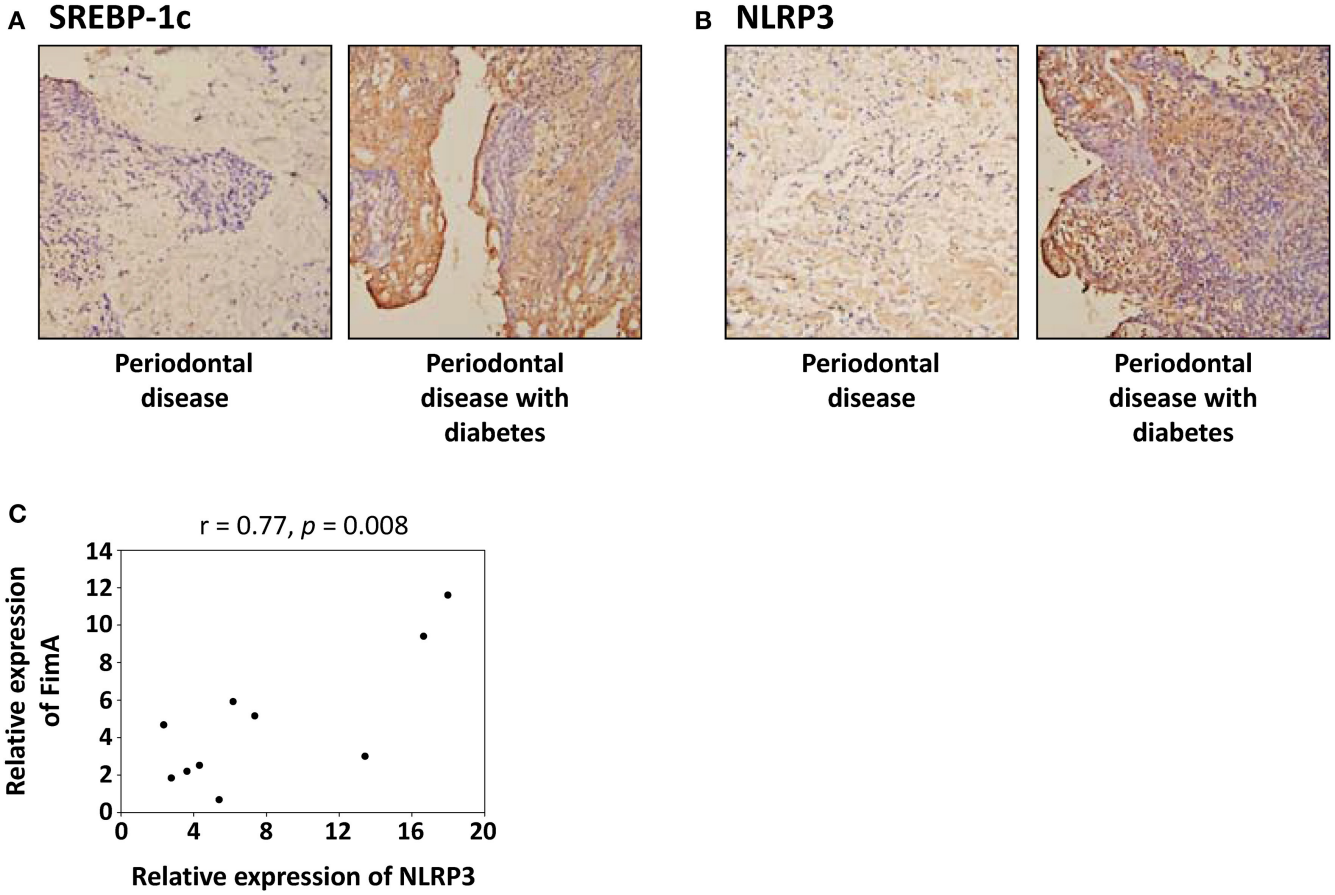
**SREBP-1c and NLRP3 expression is increased in gingival tissues from diabetic patients with periodontal disease. (A,B)** Representative images of immunohistochemical localization of SREBP-1c **(A)** and NLRP3 **(B)** in patients with periodontal disease alone and patients with both diabetes and periodontal disease. **(C)** Correlation of the relative expression level of the FimA and the relative expression level of the NLRP3 in patients with both diabetes and periodontal disease.

Figures 7A,B present respective images of periodontal tissue sections with SREBP-1c and NLRP3 immunostaining. These images show that the intensity and area of SREBP-1c (Figure 7A) and NLRP3 (Figure 7B) increased in diabetic patients with periodontal disease compared to patients with periodontal disease alone. We also performed real-time RT-PCR to detect FimA and NLRP3 gene expression in gingival samples from diabetic patients with periodontal disease. As shown in Figure 7C, the relative expression level of FimA was significantly associated with the expression level of NLRP3.

## Discussion

**Figure 8:**
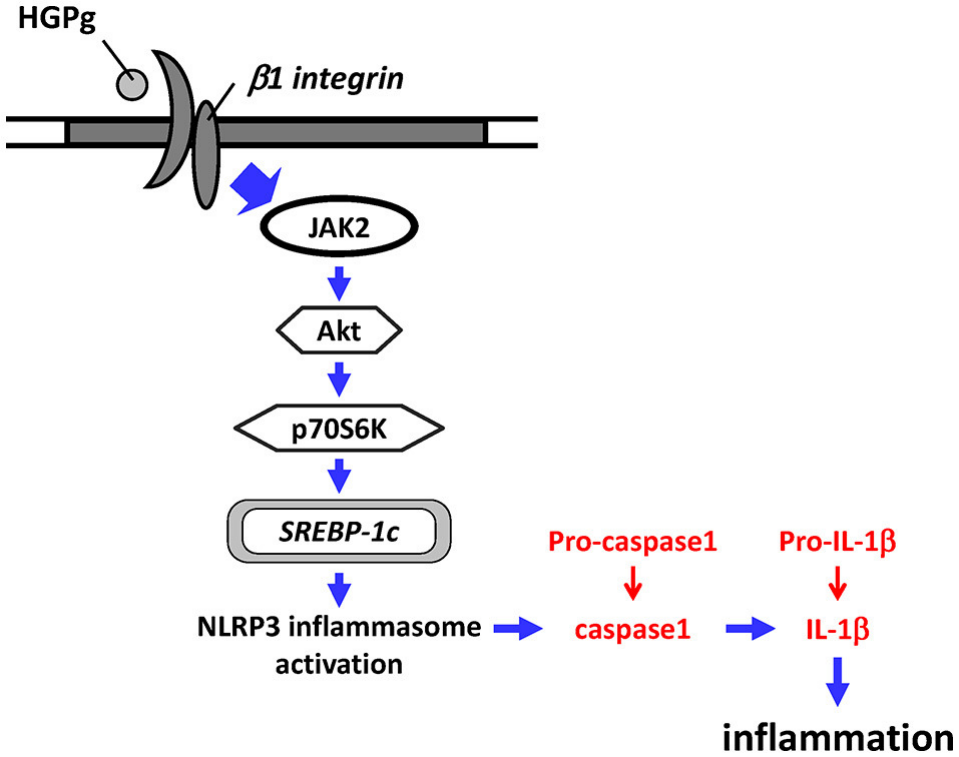
**Schematic representation of HGF NLRP3 inflammasome activation in response to HGPg infection**.

The innate immune system is regarded as the first line of defense against pathogen infection (Mogensen, 2009). The NLRP3 inflammasome complex, a key mediator of the innate immunity system, is essential for the processing of mature IL-1β via activation of caspase-1 (Jo et al., 2016). Investigating the interaction between *P. gingivalis* and host cells leading to inflammasome activation and IL-1β release is necessary to understand the development of periodontal diseases (Bostanci et al., 2009). In addition, periodontal disease seems to be associated with higher levels of inflammatory cytokines, such as IL-1β in patients with diabetes (Llambés et al., 2015). Our present study aimed to link the hyperglycemic conditions and *P. gingivalis* infection to HGF inflammasome activation and IL-1β secretion. Our results are significant in several major respects (Figure 8): (1) HGPg infection increases the expression of IL-1β and NLRP3 in HGFs compared with cells infected with NGPg; (2) HGPg-induced NLRP3 expression and mature IL-1β release is regulated by the SREBP-1c upregulation; (3) JAK2 activation and Akt/p70S6K phosphorylation are required for SREBP-1c-mediated NLRP3 and IL-1β expression; and (4) integrin β1 is a major upstream regulator for HGPg-induced signal transduction and inflammasome activation.

The presence of *P. gingivalis* and the host inflammatory response are necessary for periodontal disease development, especially in individuals with diabetes (Preshaw et al., 2012). Our previous findings demonstrated that when cultured under normal glucose conditions, NGPg has a lower FimA expression, whereas upregulated FimA expression under HG conditions and the invasion efficiency to HGFs are significantly increased by HGPg (Chang et al., 2013). In addition, *P. gingivalis* invasion is critical in the induction of host cell immune responses and the activation of inflammation-related signaling pathways (Lu et al., 2016). In this respect, the NLRP3 inflammasome plays an important role in IL-1β production in response to bacterial infection (Jo et al., 2016). The oral infection with *P. gingivalis* showed markedly increased levels of gingival NLRP3; pro-IL-1β, and pro-caspase-1 mRNA; and IL-1β protein levels compared with fimbriae-deficient mutants, suggesting that fimbriae are a virulence factor involved in NLRP3-inflammasome activation (Park et al., 2014; Yamaguchi et al., 2015). Several different cell types have been reported to express NLRP3 to process and release proinflammatory IL-1β; however, little is known about the molecular mechanisms by which HG-treated *P. gingivalis* causes IL-1β release in HGFs. In the present study, we investigated the effect of HGPg stimulation on NLRP3 expression and IL-1β production in HGFs. The results of this study demonstrate that HGPg infection not only induces NLRP3 upregulation, but also enhances active caspase-1 and mature IL-1β expression in HGFs. These data indicate that the NLRP3 inflammasome mediates IL-1β production upon HGPg infection.

SREBP-1c plays a key role in regulating the expression of genes responsible for *de novo* lipogenesis in the liver (Wang et al., 2015). In addition to examining the control of fatty acid synthesis, several studies have focused on the effect of SREBP-1c on inflammation. A previous study has shown that chronic systemic inflammation in the liver is accompanied by upregulated mRNA and protein expression of SREBP-1 *in vivo* (Zhao et al., 2015). *In vitro*, it was found that the inflammatory factors interleukin-6 and tumor necrosis factor-α raised the expression of SREBP-1c in hepatocytes (Jung and Choi, 2014). SREBP activity in endothelial cells has been previously shown to increase by stimulation with atherogenic factors (Chen Z. et al., 2014). Aberrant activation of SREBP-1 is also reported to correlate with vascular inflammatory response *in vivo*, as evidenced by the upregulation of NLRP3 inflammasome and IL-1β (Li et al., 2013). Although chronic inflammation and NLRP3 inflammasome activation have been implicated in atherosclerosis and fatty liver disease, whether SREBP-1c is involved in regulating NLRP3 and IL-1β expression in HGFs remains largely unknown. The results from the present study demonstrated that the upregulation of SREBP-1c in HGFs plays an important role in regulating NLRP3 inflammasome expression infected by HGPg. These data provide evidence that the SREBP-1c-mediated NLRP3 inflammasome activation and IL-1β production may contribute to the progression of periodontal disease under hyperglycemic conditions.

Until now, the exact mechanisms that lead to NLRP3 inflammasome activation in periodontal cells have not been fully understood. Previous studies have shown that SREBP-1c expression can be regulated through the Akt and p70S6K pathways (Laplante and Sabatini, 2010; Jeon and Osborne, 2012). The regulation of NLRP3 inflammasome activation through the Akt-dependent pathway has also been reported (Ives et al., 2015). In addition, it has been demonstrated that activation of Akt is mediated by JAK2 (Yang et al., 2014). The JAK pathway has been implicated in the control of inflammasome activation (Benoit et al., 2012). Several lines of evidence suggest that the HGPg-induced SREBP-1c and NLRP3 expression in HGFs is mediated via JAK2 activation, and via the Akt and p70S6K pathways. First, the inhibition of Akt and p70S6K phosphorylation in HGFs through pretreatment with inhibitors, or infection with DN-Akt, abolished HGPg-induced SREBP-1c and NLRP3 expression, as well as IL-1β secretion. Second, the inhibition of JAK2 activation in HGFs through transfection with specific JAK2-siRNA suppressed HGPg-induced Akt and p70S6K phosphorylation, SREBP-1c and NLRP3 expression, and IL-1β secretion. Third, when compared to patients with periodontal disease alone, the results of IHC staining also demonstrated that SREBP-1c and NLRP3 were increased in the gingival tissues of diabetic patients with periodontal disease. Based on our results, we suggest that the augmented expression of the NLRP3 inflammasome on HGFs may be involved in HGPg-associated gingival inflammation and may contribute to periodontal pathogen-mediated gingival connective tissue injury. However, the molecular details of the SREBP-1c activation of the NLRP3 inflammasome require further investigation.

Host cells need to transduce signals from cellular receptors into the interior of the cells in response to microbial invasion. A previous report has shown that some integrins interact with bacterial cell surface components, such as fimbriae, hemagglutinin, and lipopolysaccharide, in order to induce gene expression (Mysak et al., 2014). *P. gingivalis* uses the fimbriae to bind to integrin on osteoblasts and reorganize actin microfilaments to invade osteoblasts (Lamkanfi and Dixit, 2014). These investigations demonstrate a direct involvement of the integrins in the initiation of signaling in the fimbria-induced inflammatory gene expression. The present study showed that integrin β1 mediates HGPg infection-induced SREBP-1c and NLRP3 expression through the activation of JAK2 and phosphorylation of Akt and p70S6K. Since there is no experimental evidence in this study to show the interaction between integrin β1 and JAK2 in HGFs, a more detailed investigation is therefore required to better understand their relationship.

In conclusion, the present study provides information regarding the molecular basis in HGFs by which HGPg induces SREBP-1c and NLRP3 inflammasome and IL-1β expression. We found that the signaling pathways activated by infection of HGFs with HGPg are mediated by integrin β1. HGPg also induces the activation of the JAK2 and Akt/p70S6K signaling pathways, and ultimately enhances NLRP3 expression in HGFs. Our data indicate potential relevant clues regarding possible mechanisms for future therapeutic interventions.

## Author contributions

HK: Provision of study material, collection, and assembly of data and histopathological evaluation; LC: Design, collection, assembly of data, and manuscript writing; TC: Conception, collection, and assembly of data; KCL: Provision of study material or animals; KFL: Provision of study material or animals in pathology; CC: Provision of study material, collection, and assembly of data, HY: Conception and design, financial support, administrative support, manuscript writing, and final approval of manuscript. All authors read and approved the final manuscript.

### Conflict of interest statement

The authors declare that the research was conducted in the absence of any commercial or financial relationships that could be construed as a potential conflict of interest.
