# Supplementary material for: Sterol Regulatory Element-Binding Protein-1c Regulates Inflammasome Activation in Gingival Fibroblasts Infected with High-Glucose-Treated Porphyromonas gingivalis
Source: Front Cell Infect Microbiol. 2016 Dec 26;6:195. doi: 10.3389/fcimb.2016.00195 (PMC5183582; doi:10.3389/fcimb.2016.00195)
Supplement: Supplementary file 1 [file Image1.PDF]

Figure S1

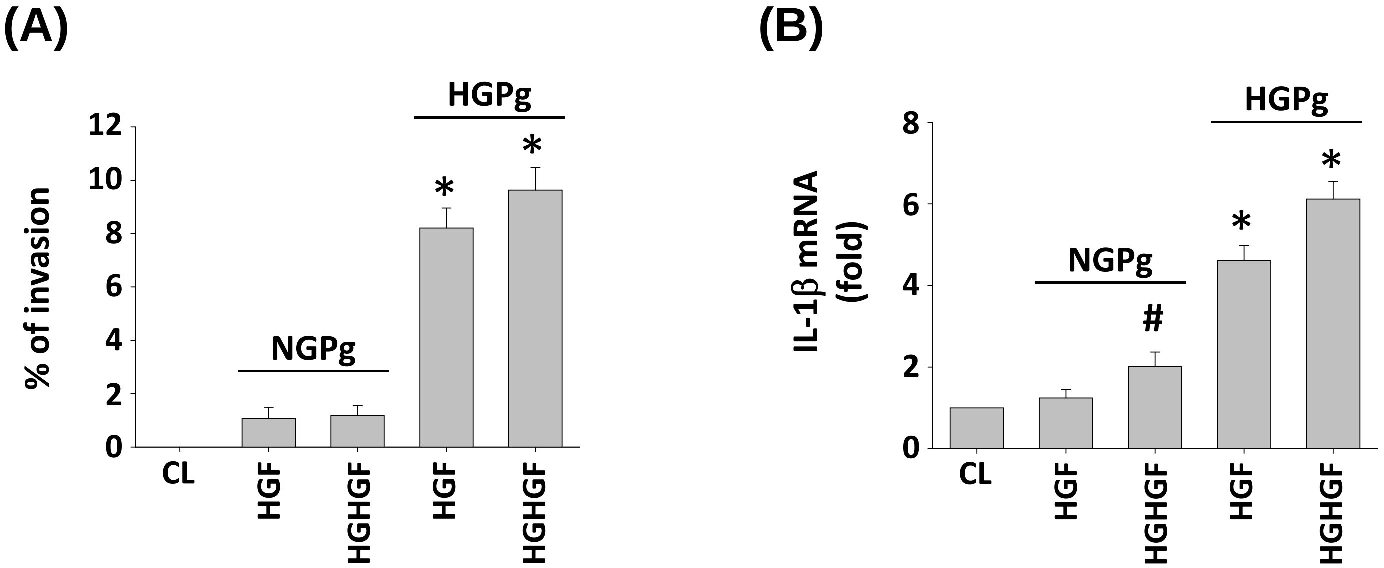

Figure S1.
(A) Invasion percentage of NGPg or HGPg to HGFs cultured under normal (HGF) or high glucose (HGHGF) conditions. *P < 0.05 versus CL or NGPg invasion.
(B) HGFs or HGHGFs were maintained as untreated controls (CL) or infected by NGPg or HGPg for 4 h. RNA samples were isolated and subjected to real-time PCR analysis. The mRNA data are presented as the fold changes compared with CL cells normalized to the 18S rRNA level. *P < 0.05 versus NGPg-infected cells. $^{\#}P$ < 0.05 versus CL cells.
