# Supplementary material for: Sterol Regulatory Element-Binding Protein-1c Regulates Inflammasome Activation in Gingival Fibroblasts Infected with High-Glucose-Treated Porphyromonas gingivalis
Source: Front Cell Infect Microbiol. 2016 Dec 26;6:195. doi: 10.3389/fcimb.2016.00195 (PMC5183582; doi:10.3389/fcimb.2016.00195)
Supplement: Supplementary file 2 [file Image2.PDF]

Figure S2

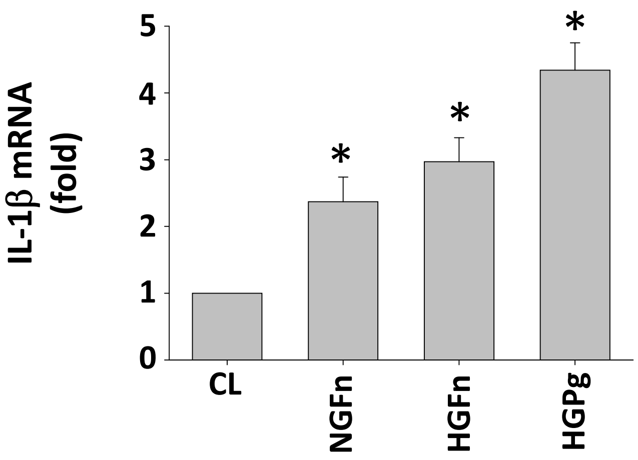

Figure S2. HGFs were maintained as untreated controls (CL) or infected by NGFn, HGFn, or HGPg for 4 h. RNA samples were then isolated and subjected to real-time PCR analysis. *$P < 0.05$ versus CL cells.
