# Supplementary material for: Sterol Regulatory Element-Binding Protein-1c Regulates Inflammasome Activation in Gingival Fibroblasts Infected with High-Glucose-Treated Porphyromonas gingivalis
Source: Front Cell Infect Microbiol. 2016 Dec 26;6:195. doi: 10.3389/fcimb.2016.00195 (PMC5183582; doi:10.3389/fcimb.2016.00195)
Supplement: Supplementary file 3 [file Image3.PDF]

Figure S3

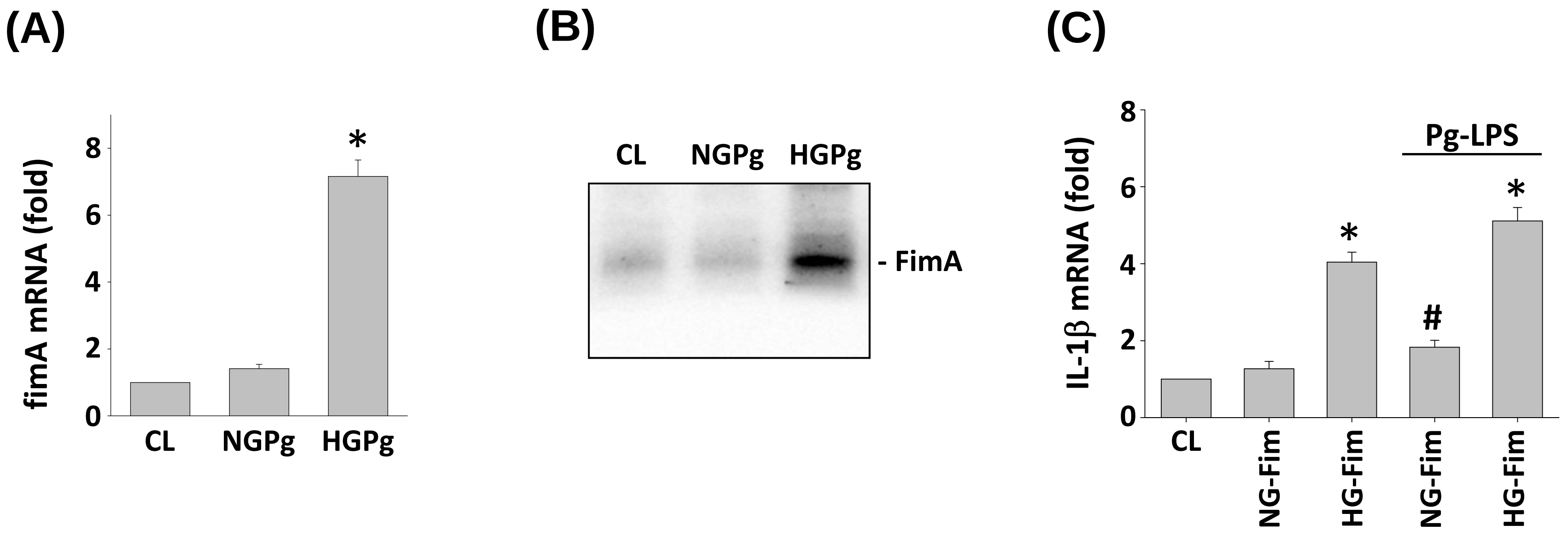

Figure S3. (A) Analysis of *fimA* mRNA expression in NGPg and HGPg. RNA samples from *P. gingivalis* were isolated and subjected to real-time PCR analysis. The mRNA data are presented as fold changes in fluorescent density from control *P. gingivalis* normalized to the 16S rRNA levels. **P* < 0.05 versus NGPg. (B) Fimbriae from 10^8 cfu/mL of NGPg and HGPg were purified and subjected to Western blot analysis. Expression of FimA were detected by using polyclonal anti-FimA antibody. (C) HGFs were grown as untreated CL or stimulated with equal volumes of purified fimbriae samples (100 μL) from 10^8 cfu/mL of NGPg (NG-Fim) or HGPg (HG-Fim), or co-stimulated with Pg-LPS and purified fimbriae for 4 h. RNA samples were then isolated and subjected to real-time PCR analysis. **P* < 0.05 versus NG-Fim-treated cells. $^{\#}P < 0.05$ versus CL cells.
